# Methodological quality and implications for practice of systematic Cochrane reviews in pediatric oral health: a critical assessment

**DOI:** 10.1186/1472-6831-14-35

**Published:** 2014-04-09

**Authors:** Violaine Smaïl-Faugeron, Hélène Fron-Chabouis, Frédéric Courson

**Affiliations:** 1 Institut National de la Santé et de la Recherche Médicale, UMR S 872, Equipe 22, Centre de Recherche des Cordeliers, Paris, France; 2 Assistance Publique-Hôpitaux de Paris, Hôpital Bretonneau, Service d’Odontologie, Paris, France; 3 Université Paris Descartes - Sorbonne Paris Cité, Faculté de Chirurgie Dentaire, Unité de Recherches Biomatériaux Innovants et Interface EA4462, 1 rue Maurice Arnoux, 92120 Montrouge, France; 4 Assistance Publique-Hôpitaux de Paris, Hôpital Charles Foix, Service d’Odontologie, Ivry-sur-Seine, France

**Keywords:** Systematic reviews, Cochrane collaboration, Children oral health, Implication for practice, Caries prevention

## Abstract

**Background:**

To ensure evidence-based decision-making in pediatric oral health, Cochrane systematic reviews that address topics pertinent to this field are necessary. We aimed to identify all systematic reviews of paediatric dentistry and oral health by the Cochrane Oral Health Group (COHG), summarize their characteristics and assess their methodological quality. Our second objective was to assess implications for practice in the review conclusions and provide an overview of clinical implications about the usefulness of paediatric oral health interventions in practice.

**Methods:**

We conducted a methodological survey including all paediatric dentistry reviews from the COHG. We extracted data on characteristics of included reviews, then assessed the methodological quality using a validated 11-item quality assessment tool (AMSTAR). Finally, we coded each review to indicate whether its authors concluded that an intervention should be implemented in practice, was not supported or was refuted by the evidence, or should be used only in research (inconclusive evidence).

**Results:**

We selected 37 reviews; most concerned the prevention of caries. The methodological quality was high, except for the assessment of reporting bias. In 7 reviews (19%), the research showed that benefits outweighed harms; in 1, the experimental intervention was found ineffective; and in 29 (78%), evidence was insufficient to assess benefits and harms. In the 7 reviews, topical fluoride treatments (with toothpaste, gel or varnish) were found effective for permanent and deciduous teeth in children and adolescents, and sealants for occlusal tooth surfaces of permanent molars.

**Conclusions:**

Cochrane reviews of paediatric dentistry were of high quality. They provided strong evidence that topical fluoride treatments and sealants are effective for children and adolescents and thus should be implemented in practice. However, a substantial number of reviews yielded inconclusive evidence.

## Background

Evidence-based dentistry has contributed substantially to improving the quality of oral health in general and in the paediatric population in particular. Systematic reviews of randomized controlled trials (RCTs) are considered the highest standard in evidence-based healthcare available to clinicians to guide clinical practice. The Cochrane Collaboration is the world’s largest producer of systematic reviews of primary research in human health care and health policy [1]. The Cochrane Oral Health Group (COHG) is one of 50 review groups within the Cochrane Collaboration.

High methodological quality is necessary for valid interpretation and application of systematic review findings [2,3]. Moreover, systematic reviews can be a source of knowledge for healthcare practice, provided that they give conclusive evidence that interventions are effective, ineffective or harmful. To our knowledge, no study has assessed the methodological quality and implications for practice of Cochrane systematic reviews of paediatric oral health.

We aimed to identify all existing systematic reviews of the COHG related to paediatric dentistry and oral health and to summarize the most relevant characteristics of the reviews. Our second objective was to evaluate the methodological quality of the systematic reviews using A Measurement Tool to Assess Systematic Reviews (AMSTAR), a validated 11-item quality assessment tool. Finally, we aimed to assess the implications for practice in the review conclusions and provide an overview of clinical implications about the usefulness of paediatric oral health interventions in practice.

## Methods

We conducted a methodological survey including all paediatric reviews indexed in the Dentistry and Oral Health category of the COHG reviews. We extracted data on characteristics of included Cochrane reviews, then assessed the methodological quality using the AMSTAR checklist. Finally, we examined the review conclusions to assess whether the experimental intervention was conclusive, ineffective, harmful or contained inconclusive evidence.

### Criteria for considering systematic reviews

Eligible systematic reviews were of paediatric dentistry and oral health produced by the COHG. In the first step, we selected systematic reviews if the authors clearly reported participants as “children” or “adolescents” in the title and objectives. If this was not clear, we systematically examined the full text of selected articles to determine whether authors defined an upper age limit as selection criteria or whether the maximum age of included patients was ≤ 18 years old. We selected updates of systematic reviews rather than initial versions. We excluded systematic reviews that included at least one RCT of adults and reviews that did not mention the age of participants.

### Search methods for systematic reviews

We identified eligible Cochrane systematic reviews indexed in the Dentistry and Oral Health category of the COHG at http://www.thecochranelibrary.com. The last search was conducted in November 2013. Two authors independently and in duplicate screened all full-text reports. Any disagreements were resolved by discussion.

### Data extraction

#### *Characteristics of included Cochrane systematic reviews*

Two reviewers extracted data independently and in duplicate, with discrepancies resolved by discussion. For each systematic review, we recorded the publication year, the country, the topic, the participants and the primary outcomes. For each meta-analysis of primary outcomes, we recorded the experimental intervention, the comparator, the number of RCTs examined, the number of participants, and the relative effect (treatment effect measure and combined estimate [95% confidence interval]).

#### *Assessment of methodological quality of Cochrane systematic reviews*

Two reviewers independently and in duplicate evaluated the methodological quality of systematic reviews using the AMSTAR checklist, a measurement tool of 11 items [4,5]. Disagreements were resolved with a third author. We did not use the PRISMA checklist because it is not intended to be a quality assessment tool as compared with the AMSTAR, which is a reliable and valid measurement tool to assess the methodological quality of systematic reviews [5,6]. The following characteristics were assessed: *a priori* design, study selection and data extraction, literature search, status of publication, characteristics of the included and excluded studies, scientific quality of the included studies, methods used to combine the findings of studies, publication bias and conflict of interest.

#### *Assessment of the implications for practice in conclusions sections of Cochrane systematic reviews*

Two reviewers independently examined the implications-for-practice paragraph in the conclusions sections of all selected systematic reviews. Disagreements were resolved by a third author. For each review, we assessed whether the experimental intervention should be implemented in practice (ie, conclusive evidence that the intervention was effective and not harmful), was ineffective and should not be used in practice, was harmful and should not be used in practice, or should be used only in research (ie, the evidence identified was inconclusive; that is, the intervention could be beneficial or harmful) [7,8]. The experimental intervention was considered ineffective if the evidence showed that it was ineffective for all primary outcomes, harmful if the evidence showed it was harmful for at least one adverse event, to be used in research only if the evidence was inconclusive for at least one primary outcome, or should be implemented in practice if the evidence showed that it was effective for all primary outcomes and not harmful, with no adverse events.

## Results

### Eligible Cochrane systematic reviews

The search yielded 278 Cochrane systematic reviews that specifically addressed dentistry and oral health issues. After 7 duplicates were removed, we finally included 37 systematic reviews focused on paediatric oral health [9-45].

### Characteristics of included Cochrane systematic reviews

The median year of publication was 2008 (range 2002–2013) (Table 1). Most systematic reviews (57%) were performed in the United Kingdom. The reviews mainly concerned interventions for the prevention of dental caries (n = 16), orthodontic treatment and oral surgery (n = 4 for each domain), treatment of dental caries (n = 3) and behavior management (n = 2). Details are given in Additional file 1: Table S1.

**Table 1:** Characteristics of included systematic reviews

**Characteristics**	**No of reviews (%)**
	**n = 37**
Publication year	
2002 – 2004	9 (24%)
2005 – 2007	8 (22%)
2008 – 2010	8 (22%)
2011 – 2013	12 (32%)
Country	
United Kingdom	21 (57%)
Brazil	3 (8%)
Germany	2 (5%)
China	2 (5%)
Ireland	2 (5%)
Finland	2 (5%)
Oman	1 (3%)
Syrian Arab Republic	1 (3%)
South Africa	1 (3%)
France	1 (3%)
The Netherlands	1 (3%)
Topic	
Prevention of dental caries	16 (43%)
Orthodontic treatment	4 (10%)
Oral surgery	4 (10%)
Treatment of dental caries	3 (8%)
Behavior management	2 (5%)
Treatment of oral pain	1 (3%)
Dental fluorosis	1 (3%)
Treatment of dental development disorder	1 (3%)
Treatment of gingivostomatitis	1 (3%)
Anesthesia	1 (3%)
Treatment of dental trauma	1 (3%)
Orthopedic treatment	1 (3%)
Craniofacial anomaly	1 (3%)

### Comparisons of primary outcomes

In 30 reviews, no meta-analysis was performed for primary outcomes in 65 comparisons: for 9 comparisons, no RCT existed for the primary outcomes; for 53 comparisons, only 1 RCT existed for the primary outcomes; and for 3 comparisons (2, 3, and 3 RCTs), no meta-analysis was performed for the primary outcomes. In 15 reviews, 65 meta-analyses were performed for primary outcomes (at least 2 RCTs included). Among the 65 meta-analyses, the median number of RCTs per meta-analysis was 3 [Q1–Q3 2–6, min–max 2–133] and the median number of patients per meta-analysis was 360 [Q1–Q3 182–1,673, min–max 50–65,179]. The number of meta-analyses with continuous outcomes was 61 (94%). Details are given in Additional file 2: Table S2.

### Methodological quality of Cochrane systematic reviews

**Figure 1:**
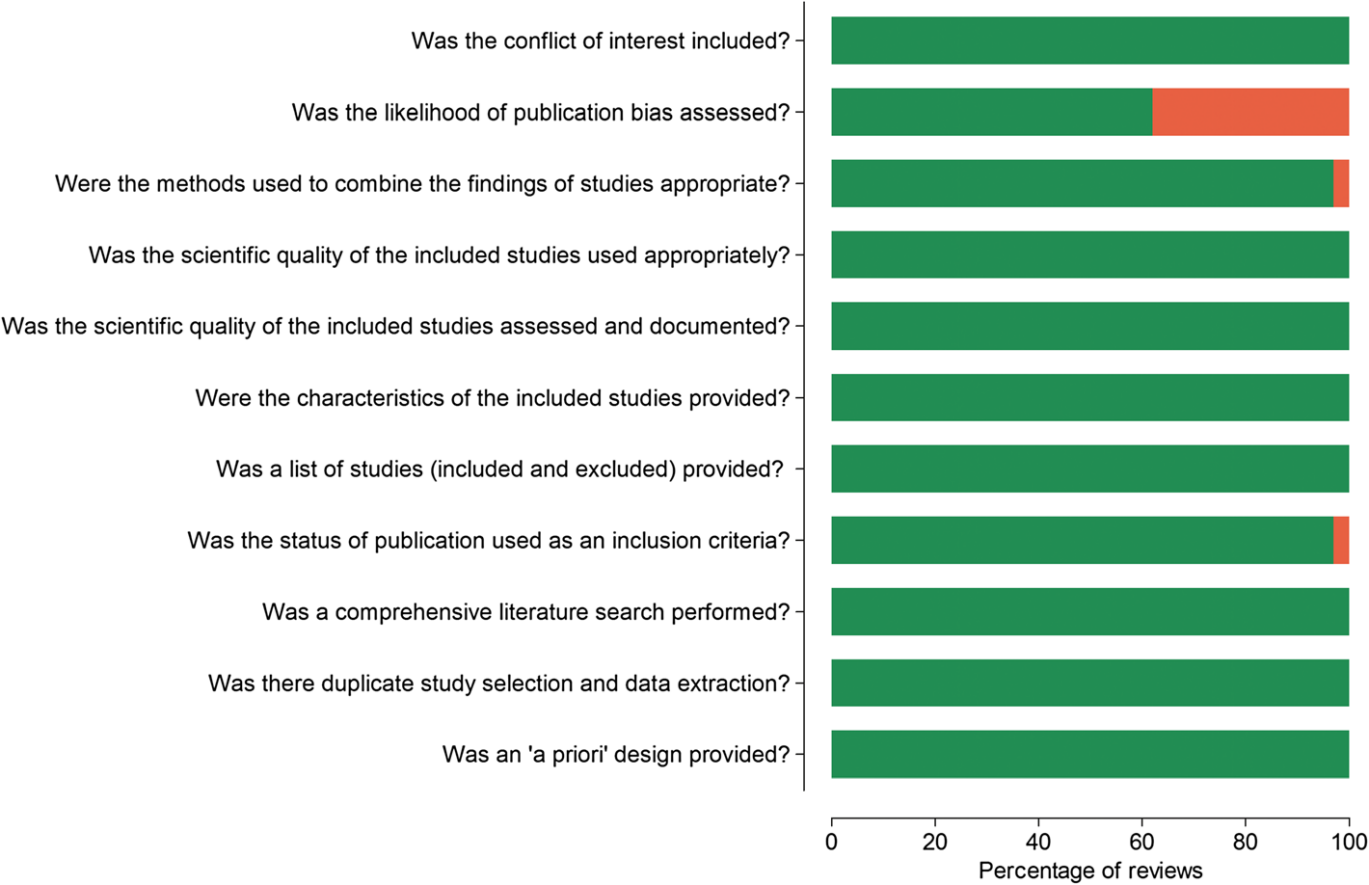
Assessment of methodological quality of included studies using AMSTAR.

The overall quality of the selected reviews was high according to the AMSTAR checklist. In all reviews, the reporting of 8 of the 11 items was adequate (Figure 1). The weakest area was failure to report the likelihood of publication bias, in 14 reviews (38%), which did not assess publication bias [11-13,17,19,20,24-26,34,35,38,42,43]. One review did not use “grey” literature as an inclusion criterion [34] and in another, the methods used to combine the findings of studies were inappropriate [38].

### Implications for practice in Cochrane systematic reviews

For the 37 systematic reviews, 7 (19%) concluded that specific interventions should be implemented in practice (ie, interventions for which research showed that benefits outweighed harms), and 1 concluded that specific interventions should not be used in practice because of ineffectiveness (Table 2). All experimental interventions that had been shown to be effective involved prevention of dental caries. Research evidence supported the effectiveness of topical fluoride treatments (with toothpaste, gel or varnish) for permanent and deciduous teeth in children and adolescents, and sealants for occlusal tooth surfaces of permanent molars. We did not identify any intervention for which research showed that harms outweighed benefits. However, for 29 reviews (78%), the evidence was inconclusive because it was limited (*see* Additional file 3: Table S3).

**Table 2:** Characteristics of systematic reviews with experimental interventions that had been found effective and should be implemented in practice

**Review**	**Participants**	**Intervention**	**Comparator**	**Outcome**	**# RCTs**	**# pts**	**Relative effect [95% CI]**
**1830**	Permanent teeth in children and adolescents	Resin-based sealant applications on occlusal tooth surfaces of permanent molars	No sealant application	Dentine caries in permanent molars Follow-up: 2 yr	6	1,066	OR 0.12 [0.07;0.19]
**2278**	Permanent teeth in children and adolescents	Fluoride toothpaste	Placebo	D(M)FS increment - nearest to 3 yr	70	42,300	SMD -0.31 [-0.35;-0.27]
**2278**	Permanent teeth in children and adolescents	Fluoride toothpaste	Placebo	D(M)FT increment- nearest to 3 yr	53	32,371	SMD -0.28 [-0.33;-0.23]
**2279**	Permanent and deciduous teeth in children and adolescents	Fluoride varnishes	Placebo/no treatment	D(M)FS increment - nearest to 3 yr	7	2,278	SMD -0.46 [-0.65;-0.26]
**2280**	Permanent and deciduous teeth in children and adolescents	Fluoride gels	Placebo	D(M)FS increment - nearest to 3 yr	14	4,492	SMD -0.20 [-0.29;-0.10]
**2280**	Permanent and deciduous teeth in children and adolescents	Fluoride gels	No treatment	D(M)FS increment - nearest to 3 yr	9	2,677	SMD -0.46 [-0.65;-0.27]
**2280**	Permanent and deciduous teeth in children and adolescents	Fluoride gels	Placebo	D(M)FT increment - nearest to 3 years	4	1,525	SMD -0.19 [-0.29;-0.09]
**2280**	Permanent and deciduous teeth in children and adolescents	Fluoride gels	No treatment	D(M)FT increment - nearest to 3 years	6	1,673	SMD -0.73 [-1.13;-0.32]
**2284**	Permanent and deciduous teeth in children and adolescents	Fluoride mouthrinses	Placebo/no treatment	D(M)FS increment - nearest to 3 years	34	14,663	SMD -0.30 [-0.36;-0.24]
**2284**	Permanent and deciduous teeth in children and adolescents	Fluoride mouthrinses	Placebo/no treatment	D(M)FT increment - nearest to 3 years	13	5,105	SMD -0.28 [-0.37;-0.20]
**2782**	Permanent and deciduous teeth in children and adolescents	Topical fluoride	Placebo/no treatment	D(M)FS increment - nearest to 3 years	133	65,179	PF 0.26 [0.23;0.29]
**2782**	Permanent and deciduous teeth in children and adolescents	Topical fluoride	Placebo/no treatment	D(M)FT increment - nearest to 3 yr	79	41,391	PF 0.26 [0.21;0.30]
**2782**	Permanent and deciduous teeth in children and adolescents	Topical fluoride	Placebo/no treatment	d(e)fs increment - nearest to 3 yr	5	1,685	PF 0.33 [0.22;0.44]
**7868**	General population of children and adolescents	Fluoride toothpaste	Placebo or other fluoride toothpaste	D(M)FS increment - nearest to 3 yr	74	Not clear	PF 19.79 [16.72;22.87]
**7868**	General population of children and adolescents	Fluoride toothpaste	Placebo or other fluoride toothpaste	D(M)FT increment - nearest to 3 yr	54	Not clear	PF 21.1 [16.86;25.47]
**7868**	General population of children and adolescents	Fluoride toothpaste	Placebo or other fluoride toothpaste	D(M)FS increment nearest to 3 yr	74	Not clear	SMD -0.24 [-0.27;-0.20]
**7868**	General population of children and adolescents	Fluoride toothpaste	Placebo or other fluoride toothpaste	D(M)FT increment - nearest to 3 yr	54	Not clear	SMD -0.24 [-0.28;-0.20]
**7868**	General population of children and adolescents	Fluoride toothpaste	Placebo or other fluoride toothpaste	d(m)fs increment - nearest to 3 yr	3	Not clear	PF 34.82 [25.68-43.96]
**7868**	General population of children and adolescents	Fluoride toothpaste	Placebo or other fluoride toothpaste	d(m)ft increment - nearest to 3 yr	3	Not clear	PF 12.18 [5.08-19.29]
**7868**	General population of children and adolescents	Fluoride toothpaste	Placebo or other fluoride toothpaste	Proportion developing new caries (permanent)	8	Not clear	RR 0.98 [0.94-1.02]
**7868**	General population of children and adolescents	Fluoride toothpaste	Placebo or other fluoride toothpaste	Proportion developing new caries (deciduous)	3	Not clear	RR 0.87 [0.81-0.93]

Yr, year; #, number; pts, participants; 95% CI, 95% confidence interval; RR: relative risk; OR: odds ratio; SMD: standardized mean difference; PF: prevented fraction = mean caries increment in controls – mean caries increment in the treated group/mean caries increment in controls. D(M)FS increment: caries increment on permanent tooth surfaces; D(M)FT increment: caries increment in permanent teeth; dmfs increment: caries increment on deciduous tooth surfaces; dmft increment: caries increment in deciduous teeth.

## Discussion

Our study shows that the number of Cochrane systematic reviews in paediatric dentistry and oral health has increased during the last few years. This situation should improve the basis for clinical decision-making because systematic reviews are considered essential sources of evidence for guideline development [46]. The methodological quality of most of our reviews was high, corresponding to the high quality standards of the Cochrane Collaboration. Nevertheless, the likelihood of publication bias was not frequently assessed. This is an important factor to take into account in the conduct of a meta-analysis and in the interpretation of results [47].

Cochrane reviews should not define recommendations for practice because this requires assumptions about the relative importance of benefits and harms of an intervention and judgements that are beyond the scope of a systematic review. However, Cochrane review authors always propose implications for practice. Our study demonstrated that most of the reviews (43%) and all interventions supported by research evidence focused on the prevention of dental caries. For children and adolescents, topical fluoride treatments (with toothpaste, gel or varnish) were found effective for permanent and deciduous teeth and sealants for occlusal tooth surfaces of permanent molars. The predominance of this topic seems justified because it is the most important from a public health policy viewpoint. Early childhood caries is the most frequent chronic disease affecting young children and is 5 times more common than asthma [48]. The selected reviews also concerned orthodontic treatment and oral surgery. However, for clinicians, several secondary research gaps are the management of oro-dental trauma or conservative treatments. Actually, the latter involve materials that may be harmful because of some toxicity [49,50].

Many of our reviews (78%) produced inconclusive evidence. The most common reasons for failure to provide reliable information to guide clinical decisions are the small numbers of RCTs and patients per meta-analysis. According to a cross-sectional descriptive analysis about characteristics of meta-analyses in the Cochrane Database of Systematic Reviews, the median number of RCTs included in meta-analyses was 3 (Q1–Q3 2–6) and the median number of patients was 91 (Q1–Q3 44–210) [51]. Our findings are consistent with these figures and emphasize that more high-quality primary research may be frequently needed to reach conclusiveness. However, none of the selected reviews was empty; that is, randomized evidence always existed and was included in the review, even when inconclusive. Another explanation for the inconclusiveness may be the inability to perform data synthesis. Diversity in outcomes measured across RCTs within a review may substantially limit the ability to perform meta-analyses and may explain the lack of recommendations [52,53]. Many meta-analyses frequently exclude a large number of RCTs because outcomes are too different between studies [54]. The standardization of outcomes was initiated by the OMERACT group [55] and is expanding with the COMET Initiative [56]. In the field of dentistry, some studies have defined core outcome sets to help solve this problem, such as in implantology [57-60] and for the evaluation of pulp treatments in primary teeth [61]. Finally, all systematic reviews should be considered as informative because they may allow for identifying well-informed uncertainties about the effects of treatments [62,63].

Previous methodological surveys assessed the conduct quality of systematic reviews in the field of dentistry [64-66]. In a study of 109 systematic reviews published in major orthodontic journals, 26 were published in the Cochrane Database of Systematic Reviews. In all, 21% of the selected reviews satisfied 9 or more of the 11 AMSTAR criteria [64,65]. However, to our best knowledge, no methodological survey concerned specifically pediatric oral health.

Our study has some limitations. Indeed, we considered only Cochrane systematic reviews in our study, but many non-Cochrane systematic reviews have also assessed interventions in the paediatric oral health field [67]. Nevertheless, Cochrane systematic reviews are the highest standard in evidence-based health care. Moreover, Cochrane reviews have a standard structure, which always includes implications for practice. Another potential limitation is that we assessed whether the experimental intervention should be used in practice, should not be used in practice or should be used only in research based on the Implications-for-practice section only and we did not critically judge the review evidence ourselves. However, Cochrane review authors describe clinical implications only after describing the quality of evidence and the balance of benefits and harms.

## Conclusions

The Cochrane reviews of paediatric dentistry and oral health were of high quality. They provided strong evidence that topical fluoride treatments and sealants are effective for children and adolescents and thus should be implemented in practice. However, a substantial number of reviews yielded inconclusive findings.

## Competing interests

The authors declare that they have no competing interests.

## Authors’ contributions

VSF drafted the manuscript. HFC and FC participated in the design of the study. All authors conceived of the study, read and approved the final manuscript.

## Pre-publication history

The pre-publication history for this paper can be accessed here:

http://www.biomedcentral.com/1472-6831/14/35/prepub

## Supplementary Material

Additional file 1: Table S1 Characteristics of included studies. Click here for file

Additional file 2: Table S2 Characteristics of review comparisons of primary outcomes. Click here for file

Additional file 3: Table S3 Assessment of implications for practice from included studies. Click here for file
