# Supplementary material for: Methodological quality and implications for practice of systematic Cochrane reviews in pediatric oral health: a critical assessment
Source: BMC Oral Health. 2014 Apr 9;14:35. doi: 10.1186/1472-6831-14-35 (PMC4108002; doi:10.1186/1472-6831-14-35)
Supplement: Additional file 1: Table S1 — Characteristics of included studies. [file 1472-6831-14-35-S1.doc]

Table A1. Characteristics of included studies

| **Review** | | **Country** | **Topic** | **Population** |
| --- | --- | --- | --- | --- |
| **1830** |  | Finland | Prevention of dental caries | Permanent teeth in children and adolescents |
| **2278** |  | United Kingdom | Prevention of dental caries | Permanent teeth in children and adolescents |
| **2279** |  | United Kingdom | Prevention of dental caries | Permanent and deciduous teeth in children and adolescents |
| **2280** |  | United Kingdom | Prevention of dental caries | Permanent and deciduous teeth in children and adolescents |
| **2284** |  | United Kingdom | Prevention of dental caries | Permanent and deciduous teeth in children and adolescents |
| **2780** |  | United Kingdom | Prevention of dental caries | Permanent and deciduous teeth in children and adolescents |
| **2781** |  | United Kingdom | Prevention of dental caries | Permanent and deciduous teeth in children and adolescents |
| **2782** |  | United Kingdom | Prevention of dental caries | Permanent and deciduous teeth in children and adolescents |
| **3067** |  | Finland | Prevention of dental caries | Permanent and deciduous teeth in children and adolescents |
| **3220** |  | United Kingdom | Treatment of dental caries | Deciduous teeth in children |
| **3315** |  | United Kingdom | craniofacial anomalies: feeding interventions for growth and development | Babies up to the age of 6 months born with cleft lip, cleft palate or cleft lip and palate |
| **3452** |  | United Kingdom | Orthodontic treatment | Children and adolescents with prominent upper front teeth |
| **3809** |  | United Kingdom | Prevention of dental caries during orthodontic treatment | Children and adolescents with fixed braces |
| **3876** |  | United Kingdom | Prevention of dental caries | General population of children |
| **3877** |  | United Kingdom | Behaviour management | Children and adolescents receiving simple treatment |
| **3879** |  | Netherlands | Oral surgery | Adolescents with impacted asymptomatic wisdom teeth |
| **4346** |  | Ireland | Prevention of oral health | General population of children and adolescents receiving dental check-ups in primary care settings |
| **4483** |  | South Africa | Treatment of dental caries | Children with tooth decay in primary teeth |
| **4621** |  | United Kingdom | Oral surgery | Children with palatally displaced maxillary canines |
| **5101** |  | United Kingdom | Prevention of dental caries | Permanent and deciduous teeth in children |
| **5512** |  | United Kingdom | Treatment of dental caries | Children with untreated tooth decay in primary molar teeth |
| **5515** |  | Brazil | Orthodontic treatment | Children and adolescents with anterior open bite |
| **5520** |  | Brazil | Orthopaedic treatment | Children and adolescents with obstructive sleep apnoea |
| **5972** |  | Ireland | Orthodontic treatment | Children and adolescents with deep bite and retroclined upper front teeth |
| **6203** |  | Brazil | Treatment of dental trauma | Children and adolescents with luxated anterior permanent teeth |
| **6334** |  | United Kingdom | Anaesthesia | Children and adolescents receiving simple treatment |
| **6700** |  | Germany | Treatment of gingivostomatitis | Children and adolescents with a diagnosis of primary herpetic gingivostomatitis |
| **6703** |  | Germany | Craniofacial anomalies: oral surgery | Children and adolescents with an established diagnosis of velopharyngeal insufficiency associated with submucous cleft palate |
| **6966** |  | United Kingdom | Oral surgery | Children and adolescents with maxillary palatally impacted canines |
| **7154** |  | Oman | Behaviour management | Children and adolescents receiving simple treatment |
| **7157** |  | Syrian Arab Republic | Treatment of dental development disorder | Children and adolescents with amelogenesis imperfecta |
| **7592** |  | France | Prevention of dental caries | General population of children and adolescents |
| **7693** |  | China | Dental fluorosis | Children under the age of 6 years at the time topical fluorides were used |
| **7868** |  | United Kingdom | Prevention of dental caries | General population of children and adolescents |
| **8050** |  | China | Craniofacial anomalies: oral surgery | Children and adolescents with unilateral cleft lip and/or palate and bilateral cleft lip and/or palate involving the alveolar process |
| **8392** |  | United Kingdom | Treatment of oral pain | Children and adolescents receiving dental treatment |
| **9378** |  | United Kingdom | Prevention of dental caries | Children |
