# Supplementary material for: Methodological quality and implications for practice of systematic Cochrane reviews in pediatric oral health: a critical assessment
Source: BMC Oral Health. 2014 Apr 9;14:35. doi: 10.1186/1472-6831-14-35 (PMC4108002; doi:10.1186/1472-6831-14-35)
Supplement: Additional file 2: Table S2 — Characteristics of review comparisons of primary outcomes. [file 1472-6831-14-35-S2.doc]

Table A2. Characteristics of review comparisons of primary outcomes

| **Review** | **Experimental intervention** | **Comparator** | **Outcome** | **No.**  **of RCTs** | **No. of participants** | **Relative effect [95% CI]** |
| --- | --- | --- | --- | --- | --- | --- |
| **1830** | Resin-based sealant applications on occlusal tooth surfaces of permanent molars | No sealant application | Dentine caries in permanent molars Follow-up: 2 yr | 6 | 1,066 | OR 0.12 [0.07 to 0.19] |
| **2278** | Fluoride toothpaste | placebo | D(M)FS increment - nearest to 3 yr | 70 | 42,300 | SMD -0.31 [-0.35, -0.27] |
| **2278** | Fluoride toothpaste | placebo | D(M)FT increment- nearest to 3 yr | 53 | 32,371 | SMD -0.28 [-0.33, -0.23] |
| **2279** | Fluoride varnishes | placebo/no treatment | D(M)FS increment - nearest to 3 yr | 7 | 2,278 | SMD -0.46 [-0.65, -0.26] |
| **2279** | Fluoride varnishes | placebo/no treatment | D(M)FT increment - nearest to 3 yr | 3 | 1,087 | No meta-analysis |
| **2280** | Fluoride gels | placebo | D(M)FS increment - nearest to 3 yr | 14 | 4,492 | SMD -0.20 [-0.29, -0.10] |
| **2280** | Fluoride gels | No treatment | D(M)FS increment - nearest to 3 yr | 9 | 2,677 | SMD -0.46 [-0.65, -0.27] |
| **2280** | Fluoride gels | placebo | D(M)FT increment - nearest to 3 yr | 4 | 1,525 | SMD -0.19 [-0.29, -0.09] |
| **2280** | Fluoride gels | No treatment | D(M)FT increment - nearest to 3 yr | 6 | 1,673 | SMD -0.73 [-1.13, -0.32] |
| **2284** | Fluoride mouthrinses | placebo/no treatment | D(M)FS increment - nearest to 3 yr | 34 | 14,663 | SMD -0.30 [-0.36, -0.24] |
| **2284** | Fluoride mouthrinses | placebo/no treatment | D(M)FT increment - nearest to 3 yr | 13 | 5,105 | SMD -0.28 [-0.37, -0.20] |
| **2780** | Fluoride varnish | Fluoride gel | D(M)FS increment - nearest to 3 yr | 1 | 254 | PF 0.14 [-0.12, 0.40] |
| **2780** | Fluoride varnish | Fluoride mouthrinse | D(M)FS increment - nearest to 3 yr | 4 | 952 | PF 0.10 [-0.12, 0.32] |
| **2780** | Fluoride varnish | Fluoride toothpaste | d(e)fs increment - nearest to 3 yr | 1 | 183 | PF -0.05 [Not clear] |
| **2780** | Fluoride toothpaste | Fluoride gel | D(M)FS increment - nearest to 3 yr | 3 | 1,256 | PF 0.00 [-0.21, 0.21] |
| **2780** | Fluoride toothpaste | Fluoride mouthrinse | D(M)FS increment - nearest to 3 yr | 6 | 2,545 | PF 0.00 [-0.18, 0.19] |
| **2780** | Fluoride gel | Fluoride mouthrinse | D(M)FS increment - nearest to 3 yr | 1 | 257 | PF -0.14 [-0.40, 0.12] |
| **2780** | Fluoride toothpaste | Other topical fluoride | D(M)FS increment - nearest to 3 yr | 9 | 3,801 | PF 0.01 [-0.13, 0.14] |
| **2781** | Fluoride toothpaste plus others (varnish, gel or rinse) | Toothpaste alone | D(M)FS increment - nearest to 3 yr | 9 | 4,026 | PF 0.10 [0.02, 0.17] |
| **2781** | Fluoride toothpaste plus others (varnish, gel or rinse) | Toothpaste alone | d(e)fs increment - nearest to 3 yr | 1 | 173 | PF 0.15 [Not clear] |
| **2781** | Fluoride mouthrinse plus gel | Fluoride mouthrinse | D(M)FS increment - nearest to 3 yr | 1 | 252 | PF 0.02 [-0.20, 0.24] |
| **2781** | Fluoride gel plus mouthrinse | Fluoride gel | D(M)FS increment - nearest to 3 yr | 2 | 497 | PF 0.23 [0.04, 0.43] |
| **2781** | Fluoride mouthrinse plus toothpaste | Fluoride mouthrinse | D(M)FS increment - nearest to 3 yr | 4 | 1,678 | PF 0.05 [-0.05, 0.15] |
| **2781** | Fluoride gel plus toothpaste | Fluoride gel | D(M)FS increment - nearest to 3 yr | 3 | 759 | PF 0.10 [-0.01, 0.21] |
| **2781** | Fluoride varnish plus toothpaste | Fluoride varnish | d(e)fs increment - nearest to 3 yr | 1 | 186 | PF 0.19 [Not clear] |
| **2782** | Topical fluoride | Placebo/no treatment | D(M)FS increment - nearest to 3 yr | 133 | 65,179 | PF 0.26 [0.23, 0.29] |
| **2782** | Topical fluoride | Placebo/no treatment | D(M)FT increment - nearest to 3 yr | 79 | 41,391 | PF 0.26 [0.21, 0.30] |
| **2782** | Topical fluoride | Placebo/no treatment | d(e)fs increment - nearest to 3 yr | 5 | 1,685 | PF 0.33 [0.22, 0.44] |
| **3067** | Sealant | Fluoride varnish | Caries yes/no at 4 yr | 1 | 226 | RR 0.42 [0.21, 0.84] |
| **3067** | Sealant | Fluoride varnish | Caries yes/no at 9 yr | 1 | 226 | RR 0.48 [0.29, 0.79] |
| **3067** | Sealant | Fluoride varnish | Caries yes/no at 12 mo | 1 | 21 | RR 0.22 [0.01, 4.06] |
| **3067** | Sealant | Fluoride varnish | Caries yes/no at 23 mo | 1 | 121 | RR 0.74 [0.58, 0.95] |
| **3067** | Sealant plus fluoride varnish | Fluoride varnish | Caries yes/no at 24 mo | 1 | 98 | RR 0.36 [0.21, 0.61] |
| **3220** | Electrosurgical pulpotomy | Formocresolpulpotomy | Teeth extracted at 12 mo | 1 | 21 | RR 3.27 [ 0.15, 72.23 ] |
| **3220** | Ferric sulphatepulpotomy | Formocresolpulpotomy | Teeth extracted at 20 mo | 1 | 70 | RR 0.0 [ 0.0, 0.0 ] |
| **3220** | Ferric sulphatepulpotomy | Root canal therapy | Teeth extracted at 2 yr | 1 | 53 | RR 4.26 [ 1.06, 17.16 ] |
| **3315** | Rigid | Squeezable bottle | Weight (kg) up to 2 mo | 1 | 101 | MD -0.05 [-0.25, 0.15] |
| **3315** | Rigid | Squeezable bottle | Weight (kg) >2 mo to 6 mo | 2 | 131 | MD -0.10 [-0.42, 0.23] |
| **3315** | Rigid | Squeezable bottle | Weight (kg) >6 mo | 2 | 130 | MD -0.15 [-0.53, 0.22] |
| **3315** | Rigid | Squeezable bottle | Length (cm) up to 2 mo | 1 | 101 | MD 0.0 [-0.84, 0.84] |
| **3315** | Rigid | Squeezable bottle | Length (cm) >2 mo to 6 mo | 2 | 131 | MD 0.20 [-0.59, 0.98] |
| **3315** | Rigid | Squeezable bottle | Length (cm) >6 mo | 2 | 130 | MD 0.21 [-0.72, 1.14] |
| **3315** | Rigid | Squeezable bottle | Head circumference (cm) up to 2 mo | 1 | 101 | MD -0.40 [-0.99, 0.19] |
| **3315** | Rigid | Squeezable bottle | Head circumference (cm) >2 mo to 6 mo | 2 | 131 | MD -0.28 [-0.70, 0.14] |
| **3315** | Rigid | squeezable bottle | Head circumference (cm) >6 mo | 2 | 130 | MD -0.66 [-1.16, -0.17] |
| **3315** | Breastfeeding | Spoon-feeding | Weight (kg) >6 mo | 1 | 40 | MD 0.47 [0.20, 0.74] |
| **3315** | Maxillary plate | No plate | Weight (kg) 2 mo (54 to 67 days) | 2 | 72 | MD -0.02 [-0.35, 0.30] |
| **3315** | Maxillary plate | No plate | Weight (kg) 6 mo (176 to 190 days) | 1 | 28 | MD -0.57 [-1.14, -0.00] |
| **3315** | Maxillary plate | No plate | Weight (kg) 12 mo (351–379 days) | 2 | 50 | MD 0.10 [-0.53, 0.73] |
| **3315** | Maxillary plate | No plate | Length (cm) up to 2 mo | 1 | 17 | MD 0.24 [-1.86, 2.34] |
| **3315** | Maxillary plate | No plate | Length (cm) >2 mo to 6 mo | 2 | 74 | MD -1.05 [-2.20, 0.11] |
| **3315** | Maxillary plate | No plate | Length (cm) >6 mo | 1 | 18 | MD -0.78 [-3.68, 2.12] |
| **3315** | Maxillary plate | No plate | Length (cm) >12 mo | 1 | 31 | MD -1.29 [-3.86, 1.28] |
| **3315** | Maxillary plate | No plate | Head circumference (cm) 3 mo | 1 | 46 | MD 0.30 [-0.66, 1.26] |
| **3315** | Maxillary plate | No plate | Head circumference (cm) 12 mo | 1 | 32 | MD 0.25 [-1.03, 1.53] |
| **3452** | Early treatment at the end of Phase I: functional | Control | Final overjet | 3 | 432 | MD -4.04 [-7.47, -0.60] |
| **3452** | Early treatment at the end of Phase I: functional | Control | Final ANB | 3 | 419 | MD -1.35 [-2.57, -0.14] |
| **3452** | Early treatment at the end of Phase I: functional | Control | PAR score | 3 | 380 | MD -12.63 [-22.28, -2.99] |
| **3452** | Early treatment at the end of Phase I: functional | Control | ANB change | 3 | 318 | MD -0.55 [-0.92, -0.18] |
| **3452** | Early treatment at the end of Phase I: headgear | Control | Final overjet | 2 | 278 | MD -1.07 [-1.63, -0.51] |
| **3452** | Early treatment at the end of Phase I: headgear | Control | Final ANB | 2 | 277 | MD -0.72 [-1.18, -0.27] |
| **3452** | Early treatment at the end of Phase I: headgear | Functional | Final overjet | 2 | 271 | MD 1.26 [-0.92, 3.44] |
| **3452** | Early treatment at the end of Phase I: headgear | Functional | Final ANB | 2 | 271 | MD -0.04 [-0.49, 0.41] |
| **3452** | Early treatment at the end of Phase I: headgear | Functional | ANB change | 2 | 284 | MD 0.01 [-0.28, 0.29] |
| **3452** | Early treatment at the end of Phase II: functional | Control | Final overjet | 3 | 343 | MD 0.24 [-0.32, 0.80] |
| **3452** | Early treatment at the end of Phase II: functional | Control | Final ANB | 3 | 347 | MD -0.03 [-0.55, 0.48] |
| **3452** | Early treatment at the end of Phase II: functional | Control | PAR score | 3 | 360 | MD 0.96 [-1.68, 3.61] |
| **3452** | Early treatment at the end of Phase II: headgear | Control | Final overjet | 2 | 238 | MD -0.24 [-0.63, 0.16] |
| **3452** | Early treatment at the end of Phase II: headgear | Control | Final ANB | 2 | 231 | MD -0.27 [-0.80, 0.26] |
| **3452** | Early treatment at the end of Phase II: headgear | Control | PAR score | 2 | 177 | MD -1.55 [-3.70, 0.60] |
| **3452** | Early treatment at the end of Phase II: headgear | Functional | Final overjet | 2 | 225 | MD -0.21 [-0.57, 0.15] |
| **3452** | Early treatment at the end of Phase II: headgear | Functional | Final ANB | 2 | 222 | MD -0.13 [-0.78, 0.53] |
| **3452** | Early treatment at the end of Phase II: headgear | Functional | PAR score | 2 | 224 | MD -0.81 [-2.21, 0.58] |
| **3452** | Adolescent treatment: functional | Control | Final overjet | 1 | 47 | MD -5.22 [-6.51, -3.93] |
| **3452** | Adolescent treatment: functional | Control | Final ANB | 2 | 99 | MD -2.27 [-3.22, -1.31] |
| **3452** | Adolescent treatment: Twin Block | Other functional appliances | Final ANB | 2 | 155 | MD -0.68 [-1.32, -0.04] |
| **3452** | Adolescent treatment: Twin Block | Other functional appliances | Final overjet | 2 | 164 | MD 0.47 [-0.12, 1.06] |
| **3809** | Acid-phosphate-fluoride mouthrinse | No mouthrinse | No. of patients with new white spots | 1 | 60 | OR 0.41 [ 0.14, 1.20 ] |
| **3809** | Stannous fluoride | MFP mouthrinse | No. of patients with new white spots | 1 | 22 | OR 0.10 [ 0.01, 1.72 ] |
| **3809** | Fluoride & antimicrobial varnish | Fluoride varnish | No. of patients with new white spots | 1 | 220 | OR 0.89 [ 0.52, 1.53 ] |
| **3809** | Compomer | GIC for banding | No. of patients with new white spots | 1 | 93 | OR 0.29 [ 0.06, 1.45 ] |
| **3809** | Fluoridated | Non-fluoridated elastics | No. of patients with new white spots | 1 | 94 | OR 0.63 [ 0.27, 1.50 ] |
| **3876** | Fluoridated milk | Non-fluoridated milk | DMFT (5 yr) | 1 | 50 | MD -0.97 [ -1.94, 0.00 ] |
| **3876** | Fluoridated milk | Non-fluoridated milk | dmft (3 yr) | 1 | 75 | MD -1.14 [ -1.86, -0.42 ] |
| **3877** | midazolam | No treatment | Mean Houpt/other behavioural score | 5 | 182 | SMD 2.98 [1.58, 4.37] |
| **3877** | N2O | Placebo | Mean Houpt/other behavioural score | 1 | 52 | SMD 0.69 [0.13, 1.26] |
| **3877** | Chloral hydrate | Chloral hydrate/hydroxyzine | Good or better behaviour | 1 | 20 | RR 0.57 [0.24, 1.35] |
| **3879** | Extraction | Retention | Quality of life after at least 5 yr follow-up | 0 |  |  |
| **4346** | Clinical examination at 12 mo | Clinical examination at 24 mo | dmfs increment (3-5 yr old) | 1 | 58 | MD -0.90 [-1.96, 0.16] |
| **4346** | Clinical examination at 12 mo | Clinical examination at 24 mo | DMFS increment (16-18 yr old) | 1 | 78 | MD -1.20 [-2.62, 0.22] |
| **4346** | Clinical examination at 12 mo | Clinical examination at 24 mo | DMFS increment (18-20 yr old) | 1 | 49 | MD -0.50 [-1.23, 0.23] |
| **4483** | restorative material | other restorative material | Pain relief | 0 |  |  |
| **4621** | Extraction of the primary maxillary canine | No treatment, delayed treatment or an alternative treatment | Prevalence of eruption or non-eruption of the permanent canine into the mouth | 2 | 128 | No meta-analysis |
| **5101** | Slow-release fluoride device | Placebo | Increase in DMFT at 2 yr compared to baseline | 1 | 63 | MD -0.72 [-1.23, -0.21] |
| **5101** | Slow-release fluoride device | Placebo | Increase in DMFS at 2 yr compared to baseline | 1 | 63 | MD -1.52 [-2.68, -0.36] |
| **5512** | Caries removal followed by placement of a preformedmetal crown | Caries removal followed by placement of a filling material or no treatment | Freedom from the main symptom of dental decay - pain | 0 |  |  |
| **5515** | Frankel’s function regulator-4 (FR-4) and lip-seal training | No treatment | Open bite correction | 1 | 40 | RR 0.02 [0.00, 0.38] |
| **5515** | Removable appliances with palatal crib associated with high-pull chincup | No treatment | Open bite correction | 1 | 60 | RR 0.23 [0.11, 0.48] |
| **5520** | Oral appliance | No treatment | Apnoea-hipopnoea index measured by polysomnography | 1 | 23 | RR 0.39 [0.20, 0.76] |
| **5972** | orthodontic braces (removable, fixed, functional) or head braces with or without extraction of permanent teeth | No treatment or delayed treatment | Dento-occlusal results of treatment, measured with the PAR index | 0 |  |  |
| **6203** | All interventions for treating luxated teeth | No treatment, delayed treatment or an alternative treatment | Pulp survival, tooth survival, function: eating, speech, pain, periodontal ligament healing, mobility | 0 |  |  |
| **6334** | Sedative agents administered via any route by an anaesthetist, dentist or other healthcare professional in any setting | General anaesthesia administered via any route by an anaesthetist, dentist or other healthcare professional in any setting | Mortality, completion of treatment, intraoperative morbidity, postoperative morbidity | 0 |  |  |
| **6700** | Acyclovir | Placebo | No. of subjects with oral lesions (8 days after administration of the intervention) | 1 | 72 | RR 0.10 [0.02, 0.38] |
| **6700** | Acyclovir | Placebo | No. of subjects in whom new extraoral lesions developed after administration of the intervention | 1 | 72 | RR 0.04 [0.00, 0.65] |
| **6700** | Acyclovir | Placebo | No. of subjects with eating difficulties (8 days after administration of the intervention) | 1 | 72 | RR 0.14 [0.03, 0.58] |
| **6700** | Acyclovir | Placebo | No. of subjects with drinking difficulties (8 days after administration of the intervention) | 1 | 72 | RR 0.11 [0.01, 0.83] |
| **6703** | Surgical procedures | No treatment or alternative treatment | Perceptual speech, Instrumental assessment, nasalance score, videonasopharyngoscopy and multiviewvideofluoroscopy assessment | 0 |  |  |
| **6966** | Surgical exposure of palatally impacted canines with an open/closed surgical technique | Untreated contra-lateral side | Gum health, bleeding on probing, recession of the gum margin, and crestal bone height | 0 |  |  |
| **7154** | Any hypnotic technique with or without any sedative agent | No hypnotic intervention or sedative agent alone | Completion of treatment, measures of behaviour between test and control groups, Difference in post-operative anxiety between test and control groups, adverse events | 3 | 69 | No meta-analysis |
| **7157** | permanent restorative materials and techniques | Alternative materials and techniques | Patient satisfaction because of reduced dental sensitivity and improved aesthetics | 0 |  |  |
| **7592** | Fluoride supplements | No fluoride supplement (placebo or no treatment) | D(M)FS increment follow-up: 24-36 mo | 3 | 1,240 | PF 0.24 [0.16;0.33] |
| **7592** | Fluoride supplements | No fluoride supplement (placebo or no treatment) | dmfs increment follow-up: 24-36 mo | 1 | 115 | PF 0.73 [0.46, 0.99] |
| **7592** | Fluoride supplements | No fluoride supplement (placebo or no treatment) | D(M)FT increment follow-up: 24-36 mo | 3 | 1,208 | PF 0.29 [0.19, 0.39] |
| **7592** | Fluoride supplements | No fluoride supplement (placebo or no treatment) | dmft increment follow-up: 24-36 mo | 2 | 696 | PF 0.46 [0.08, 0.83] |
| **7693** | Fluoride level of toothpaste: 1 550 ppm | Fluoride level of toothpaste: 1000 ppm | Fluorosis | 1 | 1,099 | RR 0.75 [ 0.57, 0.99 ] |
| **7693** | Fluoride level of toothpaste: 2440 ppm | Fluoride level of toothpaste: 1450 ppm | Fluorosis | 1 | 1,009 | RR 0.59 [ 0.44, 0.79 ] |
| **7868** | Fluoride toothpaste | Placebo or other fluoride toothpaste | D(M)FS increment - nearest to 3 yr | 74 | Not clear | PF 19.79 [16.72, 22.87] |
| **7868** | Fluoride toothpaste | Placebo or other fluoride toothpaste | D(M)FT increment - nearest to 3 yr | 54 | Not clear | PF 21.16 [16.86, 25.47] |
| **7868** | Fluoride toothpaste | Placebo or other fluoride toothpaste | D(M)FS increment - nearest to 3 yr | 74 | Not clear | SMD -0.24 [-0.27, -0.20] |
| **7868** | Fluoride toothpaste | Placebo or other fluoride toothpaste | D(M)FT increment - nearest to 3 yr | 54 | Not clear | SMD-0.24 [-0.28, -0.20] |
| **7868** | Fluoride toothpaste | Placebo or other fluoride toothpaste | d(m)fs increment - nearest to 3 yr | 3 | Not clear | PF 34.82 [25.68, 43.96] |
| **7868** | Fluoride toothpaste | Placebo or other fluoride toothpaste | d(m)ft increment - nearest to 3 yr | 3 | Not clear | PF 12.18 [5.08, 19.29] |
| **7868** | Fluoride toothpaste | Placebo or other fluoride toothpaste | Proportion developing new caries (permanent) | 8 | Not clear | RR 0.98 [0.94, 1.02] |
| **7868** | Fluoride toothpaste | Placebo or other fluoride toothpaste | Proportion developing new caries (deciduous) | 3 | Not clear | RR 0.87 [0.81, 0.93] |
| **8050** | Traditional iliac bone grafting | Artificial graft | Bone graft healing (clinical assessment) | 1 | 21 | MD -0.90 [-1.16, -0.64] |
| **8050** | Traditional iliac bone grafting | Artificial graft | Bone graft healing (radiographic assessment) | 1 | 21 | MD -0.90 [-1.39, -0.41] |
| **8050** | Traditional iliac bone grafting | Traditional iliac bone  grafting + artificial graft | Bone graft healing (radiographic assessment) | 1 | 27 | MD -42.62 [-64.25, -20.99] |
| **8392** | Paracetamol | Placebo | Presence of postoperative pain related behaviour Follow-up: mean 6.5 hr | 2 | 100 | RR 0.81 [0.53 to 1.22] |
| **9378** | Behavioural intervention (both oral hygiene and dietary components) | No intervention or delayed intervention | Caries - DMFS follow-up: 15 mo | 1 | 60 | PF 0.65 [0.12 to 1.18] |
| **9378** | Behavioural intervention (both oral hygiene and dietary components) | No intervention or delayed intervention | Plaque indices follow-up: 3 to 15 mo | 2 | 419 | SMD -0.51 [-0.80, -0.21] |

Yr, year; mo, month; 95% confidence interval; RR: relative risk; OR: odds ratio; MD: mean difference: SMD: standardized mean difference; PF: prevented fraction = mean caries increment in controls – mean caries increment in the treated group / mean caries increment in controls. D(M)FS increment: caries increment on permanent tooth surfaces; D(M)FT increment: caries increment in permanent teeth; dmfs increment: caries increment on deciduous tooth surfaces; dmft increment: caries increment in deciduous teeth; PAR: Peer Assessment Rating.
