# Supplementary material for: Methodological quality and implications for practice of systematic Cochrane reviews in pediatric oral health: a critical assessment
Source: BMC Oral Health. 2014 Apr 9;14:35. doi: 10.1186/1472-6831-14-35 (PMC4108002; doi:10.1186/1472-6831-14-35)
Supplement: Additional file 3: Table S3 — Assessment of implications for practice from included studies. [file 1472-6831-14-35-S3.doc]

**Table A3. Assessment of implications for practice from** included studies

| **Cochrane review** | **Conclusive** | **Ineffective** | **Harmful** | **Only in research** |
| --- | --- | --- | --- | --- |
| **1830** | x |  |  |  |
| **2278** | x |  |  |  |
| **2279** | x |  |  |  |
| **2280** | x |  |  |  |
| **2284** | x |  |  |  |
| **2780** |  |  |  | x |
| **2781** |  |  |  | x |
| **2782** | x |  |  |  |
| **3067** |  |  |  | x |
| **3220** |  |  |  | x |
| **3315** |  |  |  | x |
| **3452** |  | x |  |  |
| **3809** |  |  |  | x |
| **3876** |  |  |  | x |
| **3877** |  |  |  | x |
| **3879** |  |  |  | x |
| **4346** |  |  |  | x |
| **4483** |  |  |  | x |
| **4621** |  |  |  | x |
| **5101** |  |  |  | x |
| **5512** |  |  |  | x |
| **5515** |  |  |  | x |
| **5520** |  |  |  | x |
| **5972** |  |  |  | x |
| **6203** |  |  |  | x |
| **6334** |  |  |  | x |
| **6700** |  |  |  | x |
| **6703** |  |  |  | x |
| **6966** |  |  |  | x |
| **7154** |  |  |  | x |
| **7157** |  |  |  | x |
| **7592** |  |  |  | x |
| **7693** |  |  |  | x |
| **7868** | x |  |  |  |
| **8050** |  |  |  | x |
| **8392** |  |  |  | x |
| **9378** |  |  |  | x |

**Conclusive: the intervention was shown to be effective; Ineffective: the intervention was ineffective and should not be used; Harmful: the intervention was harmful and should not be used; Only in research: the intervention should be used only in research (ie, the evidence identified is inconclusive, it could be beneficial or harmful)**
